# Genomic Variance and Transcriptional Comparisons Reveal the Mechanisms of Leaf Color Affecting Palatability and Stressed Defense in Tea Plant

**DOI:** 10.3390/genes10110929

**Published:** 2019-11-14

**Authors:** Xuewen Wang, Ben-ying Liu, Qingshi Zhao, Xuemei Sun, Youyong Li, Zhifen Duan, Xinli Miao, Shan Luo, Jianbin Li

**Affiliations:** 1 College of tropic crops, Yunnan agricultural University, Puer 665000, China; qingshi.zhao@outlook.com; 2 Department of genetics, University of Georgia, Athens, GA 30602, USA; 3 Yunnan Key Laboratory of Tea Science, Menghai 666201, China; liusuntao@126.com (B.-y.L.); liusuntao2005@126.com (X.S.); liyouyong_yt@126.com (Y.L.);; 4 Tea Research Institute, Yunnan Academy of Agricultural Sciences, Menghai 666201, China; 5 School of mathematics and statistics, Chuxiong Normal University, Chuxiong 675000, China; miaoxinli@cxtc.edu.cn; 6 College of agriculture and biotechnology, Yunnan Agricultural University, Kunming 650201, China; Shanluo9981@outlook.com

**Keywords:** tea, leaf color, gene expression, anthocyanin, transcriptome, resequencing

## Abstract

Leaves are one of the most important organs of plants, and yet, the association between leaf color and consumable traits remains largely unclear. Tea leaves are an ideal study system with which to investigate the mechanism of how leaf coloration affects palatability, since tea is made from the leaves of the crop *Camellia sinensis*. Our genomic resequencing analysis of a tea cultivar ZiJuan (ZJ) with purple leaves and altered flavor revealed genetic variants when compared with the green-leaf, wild type cultivar YunKang(YK). RNA-Seq based transcriptomic comparisons of the bud and two youngest leaves in ZJ and YK identified 93%, 9% and 5% expressed genes that were shared in YK- and ZJ-specific cultivars, respectively. A comparison of both transcript abundance and particular metabolites revealed that the high expression of gene *UFGT* for anthocyanin biosynthesis is responsible for purple coloration, which competes with the intermediates for catechin-like flavanol biosynthesis. Genes with differential expression are enriched in response to stress, heat and defense, and are casually correlated with the environmental stress of ZJ plant origin in the Himalayas. In addition, the highly expressed *C4H* and *LDOX* genes for synthesizing flavanol precursors, ZJ-specific *CLH1* for degrading chlorophyll, alternatively spliced *C4H* and *FDR* and low photosynthesis also contributed to the altered color and flavor of ZJ. Thus, our study provides a better molecular understanding of the effect of purple coloration on leaf flavor, and helps to guide future engineering improvement of palatability.

## 1. Introduction

Leaf color is a critical trait of plants that affects photosynthesis, crop yield and quality. Many studies on leaf color have focused on photosynthesis [1], disease defense [2] and anthocyanin biosynthesis [3]. However, the molecular mechanism of leaf color and its effect on flavor quality in crops is insufficiently investigated. *Camellia sinensis*, a woody crop, is a good system for investigating the relationship between leaf color and palatability, since tea, the most popular beverage in the world, is made of its leaves, and tea quality is affected by leaf features. The leaf color and developmental stages determined the features of drinking tea, including taste, flavor, market and popularity. For example, young leaves are used to make the best tea, while old leaves cannot be used for drinkable tea. In addition, the tea plant is an evergreen perennial horticultural resource for landscapes. 

Breeders found a natural tea plant mutant with purple young leaves and buds in the 1980s in Puer, China, from which a high-quality drinking tea is made with a natural pink color. This tree has been selected and developed into an elite tea cultivar called Zijuan (ZJ), meaning purple tea, in 2003 through great breeding efforts. Many researchers have been interested in the tea tree color and tea flavor since the ZJ cultivar was released. The ZJ has rich flavonoids including catechins and anthocyanins, which cause healthy benefits, such as antioxidant activity and reducing lipid levels [4] and cardiovascular risk of consumers [5]. Catechins confer the stringent and characteristic taste of the tea [6]. The biosynthesis of both anthocyanins and catechins use common intermediates in the flavonoid pathway (https://www.genome.jp). Therefore, regulation on anthocyanin biosynthesis could change the contents of catechins, thus, affecting palatability through taste and flavor. Although at least 10 catechins were identified in the ZJ tea plant [7], the galloylated catechins, including epigallocatechin gallate and epicatechin gallate, are the most abundant (76%) [5]. Anthocyanins, water-soluble flavonoid compounds, contribute to the orange, red, purple and blue colors in leaves [8], fruits, i.e., grapes [9,10], and flowers [11]. Four types of anthocyanins in ZJ tea were identified, of which delphinidin-3-O-β-D-(6-(E)-p-coumaroyl) galactopyranoside and cyanidin-3-O-β-D-(6-(E)-p-coumaroyl) galactopyranoside are the major anthocyanins, accounting for 75% of total anthocyanins [12]. The purple leaves of another tea cultivar, named Wuyiqizhong18, which has a temporary purple color in the leaf next to the bud, contain higher anthocyanins, higher polyphenol, and higher carotenoid-to-chlorophyll ratio, but exhibit lower CO2 assimilation than green leaves [13]. An Isobaric tags for relative and absolute quantitation (iTRAQ) based protein analysis found that 544 proteins were changed by at least 1.5 fold between the two youngest purple leaves and the next two green leaves of ZJ. Twenty proteins of those may be involved in the anthocyanin metabolism [14]. The content of anthocyanin, not proanthocyanidins derived from anthocyanin, is much higher in the purple young leaves than green older leaves in ZJ and other green cultivars [14].

The genomic sequence of tea tree was made available recently, including a genome assembly from *C. sinensis* var. *assamica* YunKang (YK) [15] and *C. sinensis* var. *sinensis SCZ* [16]. Partial transcripts were reported in the purple leaves of tea trees. For example, a cDNA-AFLP based analysis reported different expression of some transcripts between the purple leaf and other green leaves of a tea cultivar called Wuyiqizhong18 [13]. A preliminary transcriptome analysis found 2250 differentially expressed de novo unigenes in the purple leaves of ZJ compared with its green mature leaves [17]. However, the genetic background of the ZJ tea plant is not reported. To date, the genetic variance and gene expression controlling the color transition and flavor molecules during development remains unknown.

In this study, we investigated the genetic basis and expressional regulation conferring leaf color and characteristic flavor molecules in ZJ using the tea cultivar YK as a control. YK, as one of the most planted tea cultivars, has been isolated from the same natural population as ZJ in the proposed origin place, Yunnan, of tea trees [18]. Different from existing reports, we focused on genomic variance, change of gene expression and flavor compounds in buds, the youngest leaf and the second youngest leaf, respectively, in both cultivars via combining comparative genomics, RNA-Seq technology and metabolite analysis. We aimed to identify genomic variance in ZJ and the ZJ specific expressed genes which should be responsible for the ZJ specific features when compared with YK. We further aimed to characterize expression level, splicing regulation, involved metabolite pathways of some important gene candidates for the features in ZJ. Then, we aimed at related metabolites such as catechins and anthocyanins to reveal the regulation between gene expression pattern and flavor molecules. The results elucidate the molecular mechanisms of purple color affecting flavor molecules in tea leaves, and thus, can guide breeding improvement.

## 2. Materials and Methods 

### 2.1. Materials 

Two tea plant varieties, YK with green leaves and ZJ with purple young leaves, were originally selected from the same tea population from Puer, China. These were developed to cultivar YK and ZJ by our breeders. We propagated the plants through the cuttings from the same plant at the same spot at latitude 21.9921 N, and longitude 100.4275 E in the common garden of the Yunnan tea research institute. The tea plants are ~20 years old with similar canopy sizes. Three leafy tissues (buds, the youngest leaf next to buds and the second youngest leaf next to the first leaf) were collected separately from ~200 individual trees of each tea cultivar. Samples were frozen in liquid nitrogen immediately after collecting, and kept in liquid nitrogen until subsequent RNA extraction. 

### 2.2. RNA Extraction, Sequencing and Nucleotide Sequence Accession Numbers

The RNAs from each sample were extracted using the methods as described previously [19] and sequenced with Illumina HiSeq 2500 in the paired-end 101 bp for each read. Three samples from each cultivar were sequenced and 6 G bases data were generated for each sample. The reads were archived at the SRA database of NCBI (https://www.ncbi.nlm.nih.gov) under the master accession number of Bioproject PRJNA300929 and are publicly available.

### 2.3. Transcript Assembly, Expression and Pathway Analysis

All raw short reads from the Illumina platform were checked with tool fastQC (www.bioinformatics.babraham.ac.uk/projects/fastqc/, version 0.11.4) for quality control. It was determined that the first and last 10 bases were of low quality. Then, the software Trimmomatic (version 0.33) was used to remove or trim the bad bases with parameters HEADCROP:10, CROP:90, LEADING:30, TRAILING:30, SLIDINGWINDOW:4:20 and MINLEN:50, determined by the fastQC results. Finally, we analyzed the expressed genes and/or transcripts using our previously described methods with the same parameters and scripts [20]. Briefly, we used the genome assembly (version 1) of *Camellia sinensis* var. *assamic* used as the reference [15] to guide the transcript assembling by using the cleaned RNA-Seq data with HiSAT2 (version 2.0.5) and StringTie (version 1.3.3b) [21]. We discarded the transcripts that were only supported by less than three RNA-Seq reads in total across three samples of either cultivar, and the remaining highly supported transcripts were promoted for subsequent analysis. The differential expressed genes and transcript were identified using R package DEseq2 (version 1.16) [22], using a cutoff of at least a two-fold change and *p* value < 0.001. The alternative transcript splicing was analyzed using the package Ballgown [21].

For annotation, we used the same methods described in our previous publication [19]. Briefly, novel genes that were not annotated in the published tea genome sequence [15], were searched using BLASTx with an E-value threshold of E-5 against the NR (NCBI non-redundant protein sequences), UniProtKB (Swiss-Prot and TrEMBL), KOG (euKaryotic Orthologous Groups) databases, and against the Pfam database by HMMER3 with an E-value of 1E-5. Based on the all annotation ID, gene ontology (GO) terms were retrieved from Gene Ontology database (http://www.geneontology.org/). The pathway mapping for the k number was conducted using Kyoto Encyclopedia of Genes and Genomes (KEGG) Automatic Annotation Server (KAAS, version 2.1) [23] and pathway enrichment (Hypergeometric test *p* < 0.05 and *q* < 0.05) was conducted against *Arabidopsis* and *Populus* using KOBAS (version 3.0) [24]. The transcription factor and regulator analyses were conducted by using the DEGs sequence as input against transcription factor database iTAK [25] (http://itak.feilab.net/cgi-bin/itak/index.cgi, version 17.09) based on 169 plant genome information.

### 2.4. RT-qPCR Validation of Levels of Gene Expression

RNA was extracted from each tissue of buds, the 1st, 2nd, 3rd, 4th, and 5th youngest leaves from 10–15 tea plants following the same method used for RNA-Seq. We used real-time quantitative PCR (RT-qPCR) to validate the expression level of selected interesting DEGs with primers designed against transcripts encoding sequence following the procedures described previously (Table S1). Three independent experiments were conducted. Different letters indicate significant differences in Duncan’s multiple range tests with *p* < 0.05.

### 2.5. Resequencing, Annotation of Genomic Variance and Nucleotide Sequence Accession Numbers

DNA from leaves of the variety ZJ was used to construct Illumina sequencing libraries of insert size 300–500 bp according to the standard method. The reads were cleaned with Trimmomatic V0.36 [26] following the procedures and parameters as described previously [20], and then were aligned to two released tea genome assemblies with BWA [27]. We used accurate GATK [28] to call the high quality variation via base quality score recalibration, insertion and deletion (InDel) realignment, duplicate removal, InDel and single nucleotide polymorphism (SNP) discovery using filtering parameters according to GATK Best Practices recommendations [29]. The final variants were filtered out by the read depth threshold 20 corresponding to the average genome coverage of our resequencing data and variant quality cut off at 99. The annotation of DNA variance was conducted with ANNOVAR (version 2017Jul16) [30]. The resequencing data is deposited at NCBI to be freely accessible to the public under project accession PRJNA476947 and SRA accession SRR7400801.

## 3. Results

### 3.1. Development and Features of the Purple Tea Cultivar ZJ

**Figure 1:**
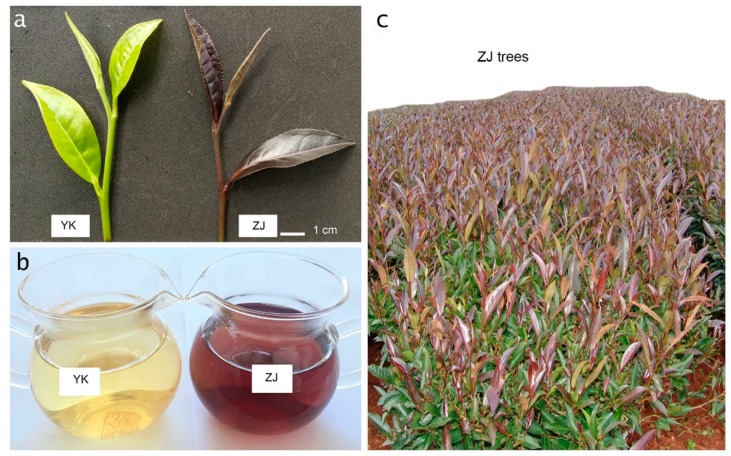
The purple features of tea cultivar ZiJuan (ZJ). Both tea cultivar YunKang (YK) and ZJ are grown at the same place and are 20 years old. (**a**) The bud, 1st and 2nd youngest leaves; (**b**) tea soup made from the tea cultivar YK and ZJ, respectively; (**c**) the canopy of the tea cultivar ZJ.

A purple tea tree was originally isolated from a natural population of 600,000 tea plants (*C. sinensis* var. *assamica*) in 1985 in Puer county, Yunnan province, China, and has been successfully developed into the novel cultivar ZJ (Figure 1a,b) with a stable purple feature after 18 years (1985-2003) of efforts by our breeders in the Yunnan Tea Research Institute. The distinct feature in ZJ from other existing tea cultivars and varieties is the purple color, present in all tender tissues, including the bud, the first three youngest leaves, and the tender shoot above the 4th youngest leaves. The fourth to fifth youngest leaves may have a mixed purple and green color. The old leaves below the 5th youngest leaves will gradually turn into the same green color as other tea varieties. The tea beverage, made from the ZJ bud and the youngest two leaves, has a natural purple or pink color, depending on the amount of leaves used and rich antioxidant compounds present, such as anthocyanins [12]. In addition, the purple canopy at the top and ever-green canopy at the bottom make ZJ one of the best candidate plants for landscapes (Figure 1c). 

### 3.2. Genetic Variance between Purple and Green Tea Variety

**Figure 2:**
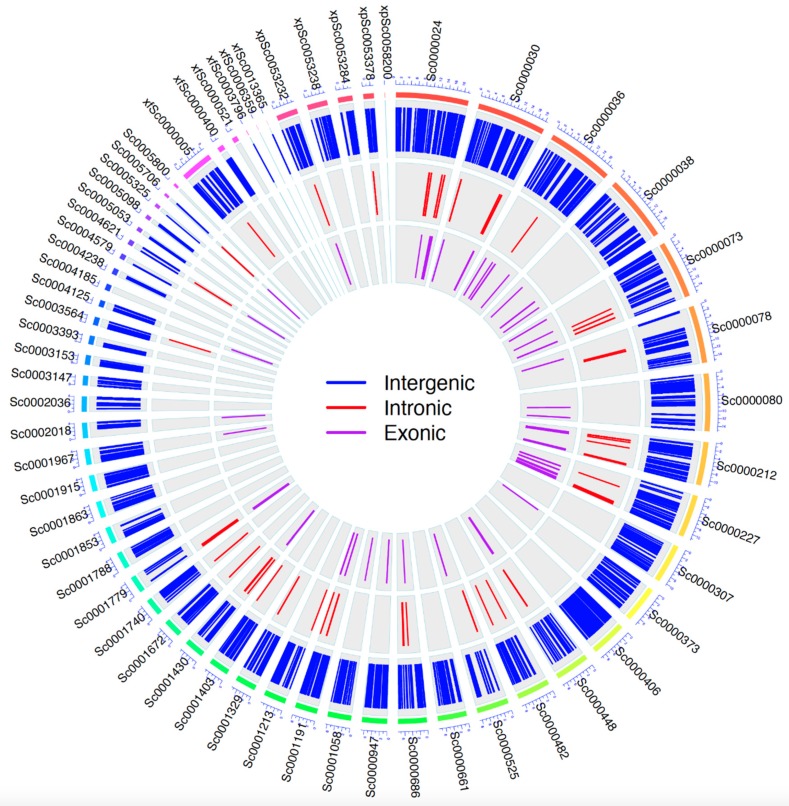
The distribution of genetic variance in the genome of purple tea cultivar ZJ. The outer circle in rainbow colors shows the scaffolds of wild type tea cultivar YK. The labels next to the outer circle are the names of the scaffolds in YK genome assembly. Three inner circles represent the variants in ZJ along the scaffolds. Each inner bar represents the position of intergenic variance (blue), or intronic variance (red) or exonic variance (purple) in purple tea cultivar ZJ.

To investigate how the genetic variance affects the gene expression, we resequenced the whole genomic DNA of purple tea variety ZJ with Illumina Xten platform. In total, 30 Gb sequence of 150-paired-end reads covering 13X of the tea genome were generated, of which 209.8 million (99.03%) were mapped back to the YK genomic assembly to detect insertions/deletions (Indels) and single nucleotide polymorphisms (SNPs). We characterized and identified 2.4 million SNPs and 675 Indels (Figure 2, Table S2). Of those, 42.7% and 4.4% sites have a read depth of 21–30X and 100X (Table S2), respectively, suggesting a high confidence of the mined variations. Most SNPs were at the intergenic regions, while only 4% SNPs were in genic regions, of which 1% and 2% were at exonic and intronic regions, respectively. At the encoded amino acid level, 58% and 39% were non-synonymous and synonymous, respectively. Interestingly, 2% SNPs resulted in gained stop frame, meaning an early termination of protein sequence at the SNP site. Of the identified InDels, 92%, 1% and 4% were at intergenic, exonic and intronic regions, respectively. Fewer SNPs and InDel (~0.1%) were observed in 3′ and 5′ UTR of genes. More information was available in Table S2. 

### 3.3. Gene Expression Profiles during Leaf Development in Purple and Green Tea Cultivar

To discover transcriptional regulation, we used RNA-Seq technology to obtain the transcriptomes and then compared them with the green tea cultivar YK. We sampled young shoot tips in the spring from ~200 20-year-old tea plants which were propagated from the cuttings of a single plant, and each collected tip had exactly a bud, the first leaf next to the bud (termed 1st leaf), the second leaf next to the first leaf (termed 2nd leaf, Figure 1a). Then, the bud, 1st leaf and 2nd leaf were separated from each tip and the same type of leaf tissue was pooled, resulting in three samples, named bud, 1st leaf and 2nd leaf. Thus, the samples could also represent different developmental stages. 

**Figure 3:**
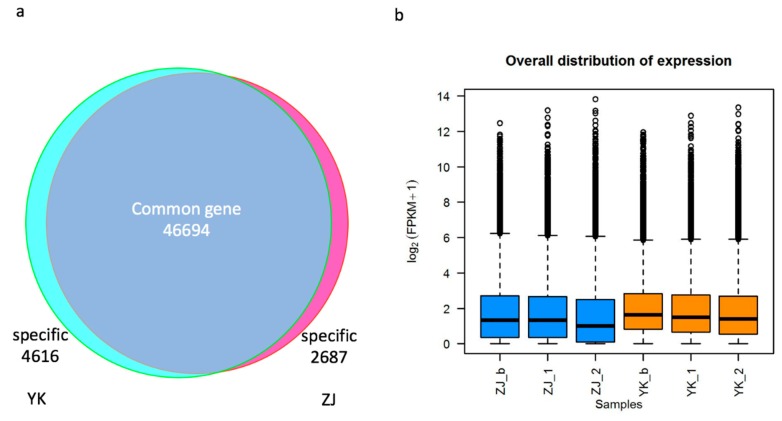
Transcriptome profiles in the purple and green tea tree. (**a**) Commonly and cultivar-specifically expressed genes across the bud, 1st and 2nd youngest leaves; (**b**) the distribution of gene expression levels in the three tissues of each cultivar. Phrase _b, _1 and _2 represent the bud, the 1st and 2nd youngest leaves of cultivar ZJ and YK.

More than 24 million, ranging from 24 to 33 million pairs of 101-bp reads were generated with Illumina HiSeq platform 2500 using RNA-Seq technology for each leaf sample of both cultivars (Table S3). 87–89% of cleaned reads were concordantly mapped to the genome assembly of YK [15] to rebuild the expressed transcripts and calculate their abundance with software Hisat2, String-tie and ballgown as described previously [21]. In total, 50,021 expressed genes and 70,010 transcripts were obtained (Table S3), which suggested more than ~20,000 novel genes were identified here than predicted 29,576 genes in the genome assembly [15]. On average, 1.4 isoforms of each gene were found in each tissue (Table S3). After discarding the genes lowly supported by ≤2 reads in all samples of each cultivar, we still got 46,694 (93%) common expressed genes in both cultivars, as well as 4616 (9%) and 2687 (5%) cultivar-specifically expressed genes in YK and ZJ, respectively (Figure 3). This suggested that less genes were expressed in purple tea ZJ, and reflected the expressional difference between YK and ZJ cultivars. 

Since the cultivar specific expression is only present in ZJ or YK, these cultivar specific genes are differentially expressed genes (DEGs) termed as specific-DEGs category here relative to common DEGs, termed as common-DEGs, expressed in both cultivars. 

To further characterize the ZJ specific-DEGs, we investigated involved metabolism pathways against the KEGG database. These specific-DEGs were enriched in three metabolism pathways, including secondary metabolites, carbon metabolism and glyoxylate and dicarboxylate metabolism (Fisher exact test, *p* < 0.05 and *q* < 0.05) (Table S4).

### 3.4. Key Specific DEGs Associated with the Purple Tea Feature

**Figure 4:**
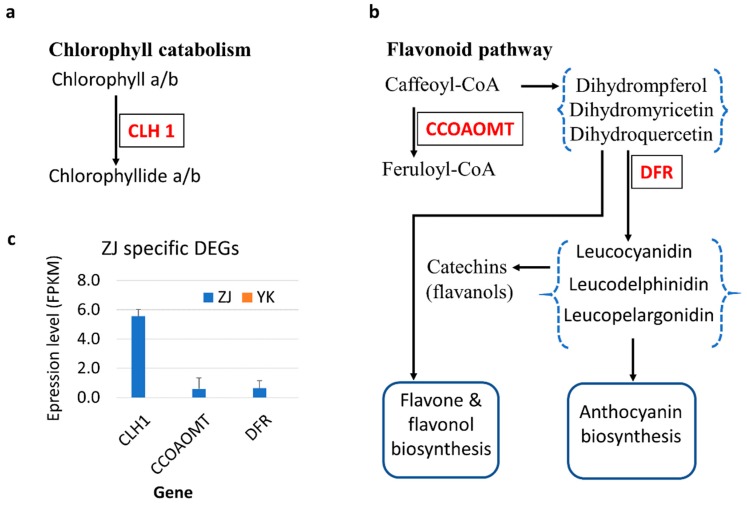
The expression and the pathways involved by the purple tea cultivar ZJ specific differentially expressed genes (DEGs). (**a**) The catabolism of chlorophyll a/b, CLH1 for chlorophyllase 1; (**b**) the flavonoid pathway, CCOAOMT: caffeoyl-CoA O-methyltransferase, DFR: dihydroflavonol 4-reductase; (**c**) the expression levels of tea cultivar ZJ specific DEGs in RNA-Seq analysis, FPKM: fragment per kilobase per million reads.

Among the ZJ specific-DEGs, we identified three genes directly associated with pigment biosynthesis or degrading. The gene N.2641 (Table S5), transcribed in only one transcript form, is annotated as an ortholog of AT1G19670 in *Arabidopsis*, encoding chlorophyllase 1 (CLH1) in the enzyme category of a carboxylic-ester hydrolase [EC: 3.1.1.14] (Figure 4a,c), which has been reported to involve in porphyrin and chlorophyll metabolism in KEGG database (ID K08099) for degrading the chlorophyll a and b during de-greening processes in plant [31]. The gene N.39992 (Figure 4b), transcribed in only one transcript form, is annotated as a homolog of AT1G24735 encoding caffeoyl-CoA O-methyltransferase (CCOAOMT) in O-methyltransferase family 3 (Interpro ID IPR002935, EC 2.1.1) belonging to the flavonoid pathway (Figure 4b,c). We identified gene N.49298 (CSA003949, Table S5), encoding dihydroflavonol 4-reductase (DFR) was expressed in one transcript form in ZJ, not in YK, in the key step of the flavonoid pathway (KEGG pathway ID ath00941, Figure 4b,c). The DFR catalyzes reactions to produce Leucocyanidin in anthocyanin biosynthesis, Leucodephidin for (high) catechins and Leucopelargonidin for (epi)afzelechin (Figure 4b). 

### 3.5. Key Pathways and DEGs among the Common Gene Set for Purple Features

**Figure 5:**
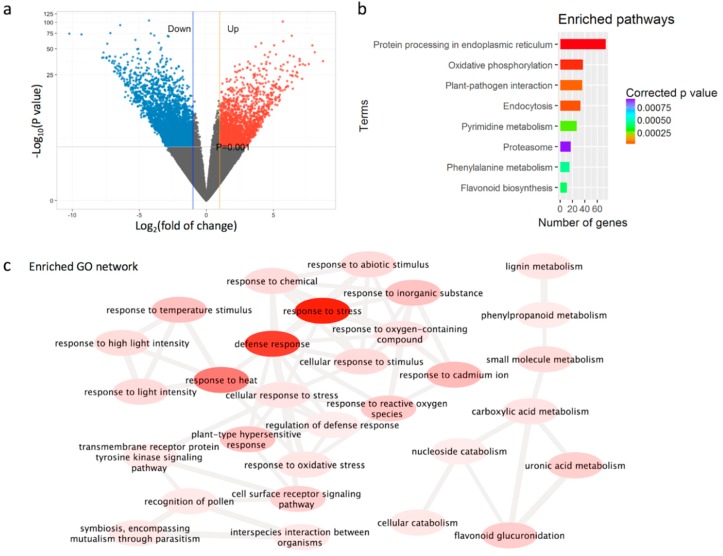
DEGs in the commonly expressed genes in leaves between green and purple tea trees. (**a**) The distribution of up- and down-regulated genes in leaves between tea cultivar ZJ and YK. The differentially expressed genes (DEGs) were shown in color with at least a two-fold change and *p* value < 0.001; (**b**) the enriched pathways with the number of DEGs; (**c**) the gene ontology network of DEGs with a color scale. The higher red indicates a higher significance with a lower *p* value.

To investigate the expressional regulation for the purple features, we examined the DEGs among the common gene set using the three leafy tissues as triple biological replicates because the gene expression for the purple feature should be present across all three tissues. In total, 1239 and 2059 genes were identified as up- and down-regulated DEGs, respectively, showing at least a two-fold change in ZJ compared with YK with DESeq2 [22] (Figure 5a, Wald test, *q* < 0.001). 

Metabolism pathway analysis revealed that these common DEGs were mapped into 110 pathways and enriched in 8 or 17 pathways in the KEGG database at corrected *p* < 0.01 or 0.05, respectively (Fishers’ exact test Table S6), including protein processing in endoplasmic reticulum, oxidative phosphorylation and flavonoid biosynthesis (Figure 5b), suggesting a serial of changes in metabolism. Further GO annotation and enrichment of the common DEGs with the tool Amigo against GO database (http://amigo.geneontology.org/amigo) revealed that the GO terms were mainly in the category of biological process and molecular function. GO enrichment and network analysis with REVIGO [32] revealed that the “response to stress”, “response to heat” and “defense response” in the biological process category were the pivotal terms (Figure 5c), while “threonine-type endopeptidase activity”, “carbohydrate derivative binding”, “ADP binding” and “threonine-type peptidase activity” in the molecular function category were the pivotal terms (Figure 5c). These results suggested that the expressional change in the purple tea ZJ was associated with stresses and defense. 

**Figure 6:**
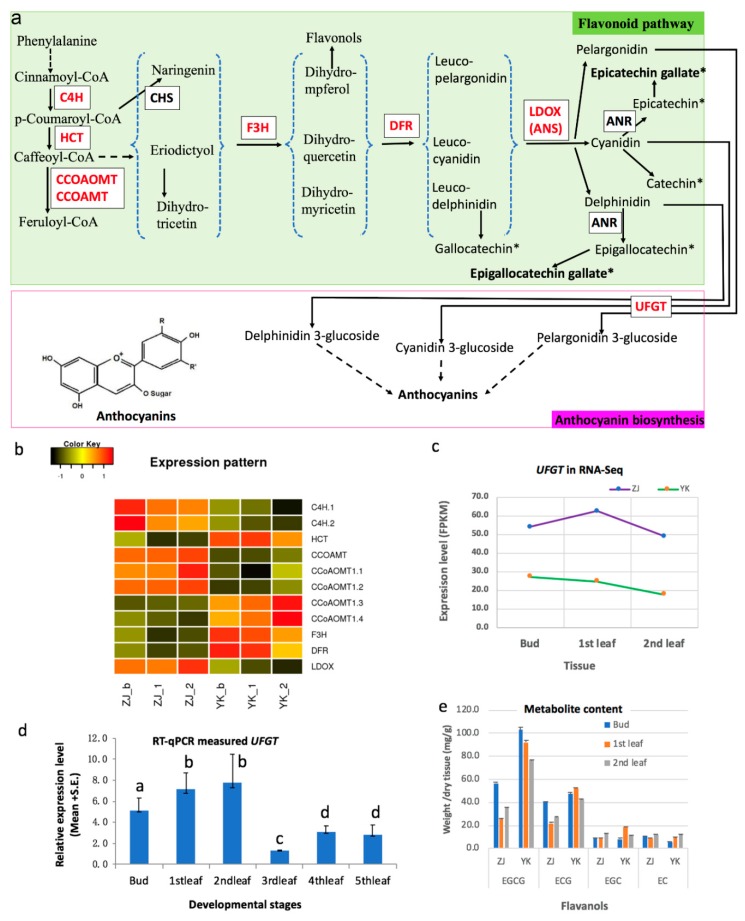
Gene expression in the flavonoid pathways and flavanols in leaves of purple tea ZJ and green tea YK. (**a**) The differentially expressed genes (DEGs) in flavonoid biosynthesis pathway, modified from KEGG map00941 and Shi et al. published review in 2014. The enzyme framed in a small box catalyzes the reaction. C4H: cinnamic acid 4-hydroxylase, CHS: chalcone synthase, F3H: flavanone 3-hydroxylase, DFR: dihydroflavanol 4-reductase, ANR: anthocyanidin reductase, UFGT: UDP-glucose: flavonoid 3-O-glucosyltransferase; (**b**) the expression levels in three tissues in tea cultivar ZJ and YK. The heatmap represents the Z-score of each gene expression after the log2 (FPKM) transformation. Suffix _b, _1 and _2 represent the bud, the 1st leaf immediately next to the bud and the 2nd leaf immediately next to the 1st leaf; (**c**) the comparison of DEG gene *UFGT* expression between two tea cultivars; (**d**) the UFGT abundance via RT-qPCR in six leafy tissues developed sequentially and letters above the bar represent the statistical significance from Duncan’s multiple range test (*p* < 0.05); (**e**) the contents of flavanols in the catechin category.

We further annotated the function of DEGs in the pathways against 29 plant species with KAAS by similarity search and then compared these DEGs expression levels between ZJ and YK. 12 DEGs were enriched in the flavonoid pathway with KEGG ID map00941, and predicted to catalyze six enzymatic steps (Figure 6a, Table 1). The abundance of transcript *C4H* in the beginning step and transcript encoding leucoanthocyanidin dioxygenase (*LDOXI*), in a late step in the flavonoid biosynthesis pathway was much higher in ZJ than YK (Figure 6b). Different expression of gene *CCOAOMT* and *CCOAMT* were found though these genes are not for biosynthesis of the precursor of anthocyanins (Figure 6a,b). Only one DEG (id N.34979, Figure 6a) was annotated to encode anthocyanidin 3-O-glucosyltransferase (UFGT) [EC:2.4.1.115, KEGG K12930] in anthocyanin biosynthesis. It is a homolog of Bronze-1 (BZ1) in maize and 3-UFGT in grape, which synthesizes glucosylate anthocyanidins in grape [9,33]. The expression pattern of *UFGT* was similar during the leaf development in both tea cultivars while the level of *UFGT* transcripts was higher in ZJ plant than YK (Figure 6c), supporting higher anthocyanins in ZJ plant [12]. In this case, we hypothesized the level *UFGT* transcripts should became lower when ZJ leaf became older and green. Our RT-qPCR measurement of *UFGT* in buds and four sequentially developed leaves proved our hypothesis (Figure 6d).

LDOX was up-regulated which catalyzes reactions to produce three shared substrates for biosynthesis of catechin-like flavanols and anthocyanins in the downstream pathways (Figure 6a). To investigate how the products go, we measured the major catechins’ content in the same leaf samples for gene expression. We found that the total catechins were lower in ZJ than YK, especially lower contents of EGCG and ECG which are most abundant catechins in tea (Figure 6e), suggesting that the product from LDOX mainly goes into the branch of anthocyanin biosynthesis and not into catechins in ZJ, consistent with previously reported higher anthocyanin in ZJ plant than other green tea cultivars [12]. The significantly up-regulated *UFGT* in the downstream may be the driven force of the LDOX product flow (Figure 6a). 

### 3.6. Low Expression of Genes in the Photosynthesis in the Purple Tea ZJ

**Figure 7:**
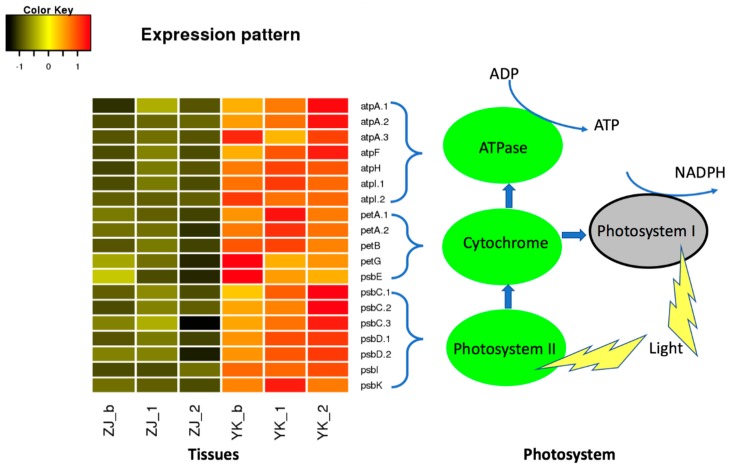
Comparison of expression levels of DEGs in photosystem in tea cultivar ZJ and YK. Image shows the differential expression levels of genes involved in the photosystem components II (green) in ZJ and YK. Suffix _b, _1 and _2 represent the bud, the 1st leaf immediately next to the bud and the 2nd leaf immediately next to the 1st leaf. The heatmap shows the Z-score of gene expression after the log2 (FPKM) transformation.

To further investigate the function of the DEGs enriched in the photosynthesis, we annotated these DEGs’ function (Table S7) against the protein sequence in the pathway database KEGG and then compared their expression levels between both cultivars. We found that 19 DEGs encode components in ATPase, cytochrome and photosystem system II in the photosynthesis pathway (Figure 7). Comparison showed that the expression of all DEGs were down-regulated in tea ZJ compared with those in YK (Figure 7), indicating a lower photosynthesis in the purple tissues, which is supported by a lower CO2 fixation rate in the purple leaf in Wuyiqizhong18 [13]. 

### 3.7. Differential Expression of Transcription Factor Genes Contributes to Features in ZJ

**Figure 8:**
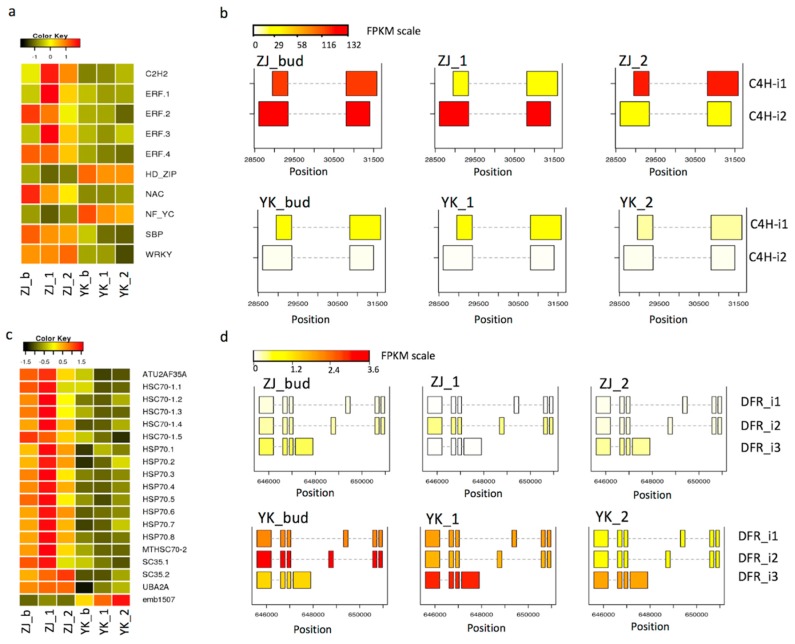
Expression of DEGs encoding transcription factors and splicing of DEGs in flavonoid pathway and spliceosome. (**a**) and (**c**) show the expression patterns of differentially expressed genes of transcription factors and the spliceosome, respectively. The heatmap shows the Z-score of gene expression after the log2(FPKM) transformation; (**b**) and (**d**) show the alternatively spliced transcripts and comparison of their expression levels in FPKM along with genomic position of gene *C4H* and *DFR,* respectively. Suffix _1 to _8 in the right side of the heatmap represent the different isoforms of the homologous gene. Suffix _b, _1 and _2 represent the bud, the 1st leaf immediately next to the bud and the 2nd leaf immediately next to the 1st leaf.

Transcription factors are known to play roles in regulation of gene expression. To understand the regulation of transcription factor, we conducted a similarity search for transcript factors in the common DEG set against the plant transcription factor database (version 4.0) and identified 10 DEGs as transcription factors, belonging into seven families (Table S8). Transcripts of eight DEGs encoding transcription factor were more abundant in ZJ than YK except HD-ZIP and NF-YC (Figure 8a). NAC and WRKY are known to be induced by stress and play roles in abiotic stresses [34,35] and metabolism [36]. Therefore, the highly expressed *NAC* and *WRKY* could function for stress response, which is consistent with our GO term enrichment into stress and secondary metabolites (Figure 5c). 

### 3.8. Alternatively Spliced DEGs in Flavonoid Pathway and DEGs in Spliceosome

To understand post-transcriptional regulation of gene expression in the flavonoid metabolism, we examined the alternative splicing (AS) in the identified DEGs. Only two genes, C4H (gene id N.41284, Table S5) and DFR (gene id N.7456, Table S5), were identified with two and three AS transcripts, respectively. Both AS transcripts, named C4H-i1 and C4H-i2, were highly present in ZJ, while only the C4H-i1 is the predominant AS in YK (Figure 8b,d), suggesting both AS transcript play roles in post-transcriptional regulation. Compared with C4H-i1, the C4H-i2 had 3′ splicing and a longer 3′ sequence. The abundance of three AS transcripts of DFR (DFR-i1, -2, -3) were higher in YK than ZJ, and the abundance of each DFR AS was dynamic with leaf development (Figure 8b,d). We further revealed that the DEGs involved in the spliceosome (Table S9), where the splicing occurs, had a distinct expression pattern. Most DEGs, especially all copies of gene *HSP70* and *HSC70*, encoding heat stress associated proteins, were highly expressed in ZJ while only the *emb1507* was highly expressed in YK (Figure 8c), suggesting a higher splicing process in ZJ. 

## 4. Discussion

The leaf is the most important plant organ for photosynthesis, energy, and secondary metabolite generation. Tea plants have become a favorite study system for linking genes to consumable traits such as flavor and/or health benefits, as tea is made from these leaves [16]. Thus, the tea cultivar ZJ is of great interest due to the purple coloration of the young leaves. Differentially expressed unigenes and protein fragments involved in phenylalanine metabolism, sugar metabolism and ATP-binding cassette transporters between the young purple leaves and old green leaves of ZJ were reported [14,17]; however, the key genetic and expressional regulatory network for the color and consumable traits remains largely unclear. In this study, we revealed several small genetic variants, of which only few cause non-synonymous changes in the gene coding regions of ZJ when compared with green wild-type YK. The majority of variants may be present in transposable elements as a major proportion of tea genome [16] or may be from naturally happened outcrossing in *Camellia* [37]. A few of these variants are worthy of further investigation. Our comparison of transcriptomes across buds, the 1st and 2nd youngest leaves between cultivar ZJ and wild-type YK reveals that the purple features mainly from the higher expression of *C4H*, *LDOX* and *UFGT* in the flavonoid biosynthesis pathway for anthocyanin; the higher expression *CLH1* for chlorophyll degradation, the lower expression of all DEGs in photosynthesis. The high expression of *LDOX* and *UFGT* genes suggests a major flow of intermediates into anthocyanin biosynthesis rather than into the catechin-category flavanols, which is the key driving force for the high accumulation of anthocyanins conferring purple coloration. Thus, the competition within the divergent pathways of shared intermediates is directly linked with flavor molecule catechins and leaf coloration molecules in tea. 

To our best knowledge, this is the first report of enriched function of DEGs in purple tissues of ZJ plant being associated with stress response and defense. This could be the result of regulation of anthocyanins. Anthocyanins have been known for their association with color change, enhanced stress response to light, temperature and heat [3]. For example, the gene Sb06g029550, which encodes a flavanone 4-reductase in the 3-deoxyanthocyanidin biosynthesis pathway, is responsible for leaf coloration change during wounding in Sorghum [38]. The role of anthocyanin has been known to increase the defense against stresses [3,11] and anthocyanin is higher in ZJ than in other green tea plants [12]. The potential multiple genetic variants in the purple tea ZJ may lead to the expressional change which increases flavonoids (including anthocyanin content) to improve stress defense and adaptation to low photosynthesis. Major secondary metabolites affecting tea taste are produced in the flavonoid pathway [16]; therefore, the regulation of genes encoding the last two key enzymes for intermediates before flavanols and anthocyanins could affect tea palatability. Of course, other DEGs beside the flavonoids in our study could contribute into the enrichment of response to stress. For example, stress related proteins such as glutathione S-transferases and phospholipid hydroperoxide glutathione peroxidase which differ between tender and older leaves of the tea Wuyiqizhong 18, which shows transitional purple color [39]. Here, we provide additional insights into the molecular network of the purple color and flavor in purple tea ZJ. 

The purple coloration in leaves is developmentally dependent, meaning its presence in the young tissues disappears gradually with maturation. Therefore, common gene expression investigated across these three young purple tissues is represented by the three ideal replicates in ZJ and YK. We examined the statistically differentially expressed genes shared by all the purple stages after comparing them with the same tissues in wild-type YK to eliminate the developing effects. Thus, these results are more robust than previous studies, which were based on de novo transcript assemblies and are simple comparisons of purple tea leaves and green tea leaves without consideration of the developmental stage. The strength of our study is demonstrated by the fact that we narrowed down DEGs associated with purple color to *C4H*, *LDOX* and *UFGT*, which is in line with the long list of previously identified candidate genes [14,17]. 

The *UFGT* gene may be the most important gene for both purple color and flavor. The expression of A-type *UFGT* has ben positively correlated with glycosylated flavonols (tea flavor molecules), i.e., F-glycosides Glc and Gal, which are higher in the buds of some tea cultivars [40]. The higher expression of *UFGT* could lead to a higher level of UFGT protein, which is evidenced by protein analysis in ZJ [14]. Thus, the high expression of *UFGT* in ZJ leads to the higher level of flavonoids, demonstrated by, namely, anthocyanins [14] and glycosylated flavonols [40]. Here, no difference was found in transcripts relating to the synthesis of proanthocyanin, which suggests that the anthocyanin is mainly accumulated and is not converted to derived proanthocyanin. In addition, the *UFGT* transcript level here is not correlated with other compounds in the catechin category, i.e., EGCG and ECG. Together, we conclude that expressional up-regulation of the gene *UFGT* could increase glycosylated flavonols (tea flavor molecules) and anthocyanins, but those are not the flavanols in the catechin category.

Chlorophyll degradation will be an additional explanation for purple color in ZJ tea plant. After comparing with the wild type YK, we initially identified the ZJ specifically expressed gene *CLH1* associated with chlorophyll degradation, which indicated low chlorophyll levels in the young purple leaves of ZJ. This was supported by the observation of lower chlorophyll content in purple leaves when compared with mature green leaves in ZJ [4]. Therefore, *CLH1*, which is cultivar-specific to ZJ, also contributes to the observed purple coloration. Since the older, greener leaves of ZJ have higher chlorophyll content, the high expression of *CLH1* may be specific to young tissues and is therefore controlled by the developmental stage. Low chlorophyll may also explain the observed low expression of genes in photosynthesis, while higher anthocyanin content may increase adaptation of young leaves, especially for the severe conditions of the high Himalayas near to the origin place of ZJ plant. 

Both transcriptional and post-transcriptional regulation contribute to the features in the purple tea ZJ. At the transcriptional level, transcription factors are known to regulate the gene expression. We identified differentially regulated transcription factors in young, purple leaves of ZJ when compared with green young leaves of YK. The transcription factors WRKY, NAC and HD-ZIP are known to be involved in stress responses [34]; therefore, the differentially expressed transcription factors identified here could regulate these DEGs in stress responses. At the post transcription level, we found evidence of alternative splicing of gene *C4H* and *DFR* in the flavonoid biosynthesis pathway and differential expression of genes encoding spliceosomal components. The alternatively spliced transcript is in low abundance in most cases; however, we found that all AS transcripts were highly abundant, indicating important regulation and potential function of all AS transcripts in regulation of flavor molecules. 

In conclusion, we analyzed the genetic variants, RNA-Seq profiles, and metabolites in buds and the two youngest leaves, and revealed key genes regulating both color and flavor molecules in ZJ compared with its wild-type relative YK. The genetic variants are the basis for altered gene expression and adaptation to environmental stress. High expression of *CLH1, C4H*, *LDOX* and especially *UFGT* leads to the high anthocyanin concentration which confers purple coloration and low concentration of catechin-category flavonoids. Low photosynthesis rates and alternative transcript splicing may be responsible for stress defense. This study has improved our understanding of the mechanism of relationship of purple coloration and its association with consumable flavor molecules in tea leaves. Thus, these results may also potentially guide the engineering of purple features and flavors of tea.

## Figures and Tables

**Table 1:** Annotation of the differentially expressed genes in the flavonoid biosynthesis pathway.

Gene_ID	KEGG k Number	Gene_Name	Enzyme	Homologous in *Arabidopsis*
N.41284	K00487	C4H.1	cinnamate-4-hydroxylase	AT2G30490
N.41281	--	C4H.2	cinnamate-4-hydroxylase	AT2G30490
N.37500	K13065	HCT	hydroxycinnamoyl transferase	AT5G48930
N.26308	K13067	CCOAMT	caffeoyl-CoA 3-O-methyltransferase	AT1G67980
N.25044	K00588	CCoAOMT1.1	caffeoyl-CoA O-methyltransferase	AT1G24735
N.18985	K00588	CCoAOMT1.2	iron-sulfur protein required for NADH dehydrogenase	AT4G19540
N.25792	K00588	CCoAOMT1.3	caffeoyl-CoA O-methyltransferase	AT4G34050
N.41150	--	CCoAOMT1.4	caffeoyl-CoA O-methyltransferase	AT4G34050
N.8528	--	F3H	flavanone 3-hydroxylase	AT3G51240
N.7456 (CSA035727)	K13082	DFR	dihydroflavonol 4-reductase	AT5G42800
N.24974 (CSA011508)	K05277	LDOX or ANS	leucoanthocyanidin dioxygenase	AT4G22880
N.34979	K17193	UFGT	UDP-glucose: flavonoid 3-O-glucosyltransferase	AT5G54060

## Supplementary Materials

The following are available online at https://www.mdpi.com/2073-4425/10/11/929/s1, Table S1: Primers for RT-qPCR, Table S2: Genetic variation in the purple tea ZJ detected by whole genome resequencing, Table S3: Summary of RNA-Seq data, genes and transcripts, Table S4: Metabolism pathways involved by the purple tea plant ZJ specific-DEGs, Table S5: Gene position in Tea genome assembly, Table S5: The pathways enriched by the DEGs, Table S6: The pathways enriched by the DEGs, Table S7: Annotated DEGs function in photosynthesis pathway, Table S8: Genes encoding transcription factors identified from the in common DEGs, Table S9: The differentially expressed genes and their annotation in the spliceosome.
