# Supplementary material for: Genomic Variance and Transcriptional Comparisons Reveal the Mechanisms of Leaf Color Affecting Palatability and Stressed Defense in Tea Plant
Source: Genes (Basel). 2019 Nov 14;10(11):929. doi: 10.3390/genes10110929 (PMC6895910; doi:10.3390/genes10110929)
Supplement: Supplementary file 1 [file genes-10-00929-s001.pdf]

Supplementary information

Genomic variance and transcriptional comparisons reveal the mechanisms of leaf color affecting palatability and stressed defense in tea plant

Xuewen Wang[1,2+*], Ben-ying Liu[3,4+], Qingshi Zhao[1+], Xuemei Sun[3,4+], Youyong Li[3,4], Zhifen Duan[3,4], Xinli Miao[5], Shan Luo[6], Jianbin Li[1*]

Table S1 Primers for RT-qPCR 1
Table S2 Genetic variation in the purple tea ZJ detected by whole genome resequencing 1
Table S3 Summary of RNA-Seq data, genes and transcripts 2
Table S4 Metabolism pathways involved by the purple tea plant ZJ specific-DEGs 3
Table S5 Gene position in Tea genome assembly 3
Table S6 The pathways enriched by the DEGs 5
Table S7 Annotated DEGs function in photosynthesis pathway 7
Table S8 Genes encoding transcription factors identified from the in common DEGs 7
Table S9 The differentially expressed genes and their annotation in the spliceosome 8

Table S1 Primers for RT-qPCR

Gene	Name	Primer sequence	Product size (bp)	Tm
UFGT	UFGT2691R	TGAAGACTCCACCCTCAACTC	344	59
	UFGT2347F	AACCGACACCTGATGAGTTGG		
GAPDH	refR	CAGTGGGAACACGGAAAGC	210	59
	refF	TTGGCATCGTTGAGGGTCT		

Table S2 Genetic variation in the purple tea ZJ detected by whole genome resequencing

Variance		SNP		InDel	
		Count	Percent	Count	Percent
Category	exonic	32,463	1%	675	1%
	exonic and splicing (overlapped)	2	0%	0	0%

	splicing region	339	0.01%	14	0.02%
	3' UTR	1,086	0.04%	83	0.1%
	5' UTR	520	0.02%	61	0.1%
	intronic	42,958	2%	2,638	4%
	upstream of gene	12,940	1%	673	1%
	downstream of gene	14,474	1%	729	1%
	upstream + downstream (overlapped)	1,373	0.1%	80	0.1%
	intergenic	2,341,433	96%	57,139	92%
	sub-total	2,447,588	100%	62,092	100%
Effect of variance					
	Synonymous SNV	12,710	39%	--	
	Nonsynonymous SNV	18,948	58%	--	
	Frameshift deletion	--		276	41%
	Frameshift insertion	--		163	24%
	Nonframeshift deletion	--		138	20%
	Nonframeshift insertion	--		86	13%
	stopgain	741	2%	10	1%
	stoploss	66	0.2%	2	0.3%
	sub-total	32,465	100%	675	100%

Phrase “NA” represents not applicable.

Table S3 Summary of RNA-Seq data, genes and transcripts

	Tea cultivar YK			Tea cultivar ZJ		
Sample	YK-b	YK-1	YK-2	ZJ-b	ZJ-1	ZJ-2
Read pairs	23,551,472	24,804,094	29,478,828	32,800,477	31,493,582	32,663,567

Kept reads (%)	89.06%	86.31%	85.81%	87.57%	86.98%	87.17%
Mapping ratio	86.68%	89.19%	87.23%	89.10%	88.14%	89.39%
Expressed genes	50,021	49,700	49,469	48,395	48,247	45,907
Transcripts	70,010	69,608	69,753	67,261	66,990	63,008
isoform/gene	1.4	1.4	1.4	1.4	1.4	1.4
novel genes	20,445	49,700	49,469	48,395	48,247	45,907
novel transcripts	40,434	69,608	69,753	67,261	66,990	63,008
Common genes in YK and ZJ			46,694			
Common transcripts in YK and ZJ #			79,718			
Specific genes in YK#			4,616			
Specific genes in ZJ#			2,689			
Predicted genes in genome assembly			29,576			

# represent the genes or transcripts were kept only if they were supported by > 2 pairs of reads to increase the confidence.

Table S4 Metabolism pathways involved by the purple tea plant ZJ specific-DEGs

KEGG pathway term	ID	Input number	Background number	p-Value	q-Value
Biosynthesis of secondary metabolites	ath01110	72	1076	8E-05	3E-03
Carbon metabolism	ath01200	23	262	1E-03	0.02
Glyoxylate and dicarboxylate metabolism	ath00630	10	74	2E-03	0.03

Table S5 Gene position in Tea genome assembly

Transcript_ID	Gene_name	Scaffold	Start_position	End_position	Length(bp)
N.1027.1	N.1027	Sc0000024	1744367	1745196	830
N.10288.1	N.10288	Sc0000482	534018	535901	1884
N.10935.1	CSA011997	Sc0000525	133102	138255	5154
N.1231.1	N.1231	Sc0000030	1625324	1626825	1502
N.12825.1	CSA020312	Sc0000661	506671	507782	1112

N.13154.1	N.13154	Sc0000686	468470	469509	1040
N.1400.1	CSA027456	Sc0000036	215992	220515	4524
N.1471.1	CSA016462	Sc0000038	1567860	1570406	2547
N.16335.1	N.16335	Sc0000947	451043	451768	726
N.17549.1	CSA000306	Sc0001058	280090	283347	3258
N.18985.1	N.18985	Sc0001191	126771	129559	2789
N.19258.1	N.19258	Sc0001213	170746	171850	1105
N.20383.1	N.20383	Sc0001329	26488	26761	274
N.21083.1	CSA031324	Sc0001403	496559	497727	1169
N.21333.1	CSA015350	Sc0001430	50227	50566	340
N.21334.1	CSA015352	Sc0001430	68621	70066	1446
N.23536.1	N.23536	Sc0001672	355386	355787	402
N.24172.1	N.24172	Sc0001740	120730	121007	278
N.24424.1	CSA003395	Sc0001779	213178	216115	2938
N.24492.1	CSA011152	Sc0001788	123086	129174	6089
N.2454.1	CSA021849	Sc0000073	815929	824178	8250
N.24974.1	CSA011508	Sc0001853	326002	327929	1928
N.25044.1	N.25044	Sc0001863	1809	4507	2699
N.25443.1	N.25443	Sc0001915	292966	293525	560
N.25792.1	N.25792	Sc0001967	290813	291616	804
N.2591.1	CSA031667	Sc0000078	485678	492363	6686
N.26157.1	N.26157	Sc0002018	98803	99747	945
N.26308.1	N.26308	Sc0002036	373568	374415	848
N.2641.1	N.2641	Sc0000080	1142520	1142747	228
N.33054.1	N.33054	Sc0003147	255170	257204	2035
N.33080.1	N.33080	Sc0003153	222473	223119	647
N.34220.1	N.34220	Sc0003393	77544	78223	680
N.34979.1	N.34979	Sc0003564	54198	57064	2867
N.37107.1	CSA013672	Sc0004125	100825	101143	319
N.37317.1	CSA009227	Sc0004185	32966	36954	3989
N.37500.1	N.37500	Sc0004238	33784	35088	1305
N.38609.1	N.38609	Sc0004579	22656	23644	989
N.38768.1	N.38768	Sc0004621	56813	57100	288
N.39878.1	N.39878	Sc0005053	19364	19843	480
N.39992.1	N.39992	Sc0005098	23984	41887	17904
N.40468.1	N.40468	Sc0005325	35063	40945	5883
N.41150.1	N.41150	Sc0005706	71108	71857	750
N.41281.1	N.41281	Sc0005800	17893	18094	202
N.41284.1	N.41284	Sc0005800	28608	31404	2797
N.42727.1	CSA010605	xfSc0000005	52100	55798	3699
N.44348.1	N.44348	xfSc0000400	27311	28387	1077

N.44638.1	CSA000963	xfSc0000521	60518	63961	3444
N.46900.1	N.46900	xfSc0003796	13150	13720	571
N.47270.1	N.47270	xfSc0006359	3672	4772	1101
N.47692.1	N.47692	xfSc0013365	246	1653	1408
N.48982.1	N.48982	xpSc0053232	450922	453062	2141
N.49039.1	CSA013761, CSA013762	xpSc0053238	411229	463245	52017
N.49298.1	CSA003949	xpSc0053284	44851	45209	359
N.49749.1	N.49749	xpSc0053378	77088	77526	439
N.53682.1	N.53682	xpSc0058200	1303	8011	6709
N.5683.1	N.5683	Sc0000212	1070387	1071396	1010
N.5684.1	CSA006934	Sc0000212	1074608	1074965	358
N.5685.1	CSA006923	Sc0000212	1075587	1075797	211
N.5989.1	CSA023075	Sc0000227	1054384	1060380	5997
N.7456.1	CSA035727	Sc0000307	645589	650972	5384
N.8528.1	N.8528	Sc0000373	455664	465863	10200
N.9047.1	CSA035886, CSA035887	Sc0000406	819026	828276	9251
N.9727.1	CSA029028	Sc0000448	66544	76723	10180

Table S6 The pathways enriched by the DEGs

Term	KEGG pathway ID	Input number	Background number	P Value	Corrected P Value
Protein processing in endoplasmic reticulum	ath04141	74	212	4E-21	6E-19
Metabolic pathways (all)	ath01100	231	1910	3E-08	2E-06

Oxidative phosphorylation	ath00190	37	162	4E-07	1E-05
Endocytosis	ath04144	33	142	1E-06	4E-05
Plant-pathogen interaction	ath04626	36	167	2E-06	6E-05
Pyrimidine metabolism	ath00240	27	116	1E-05	3E-04
Flavonoid biosynthesis	ath00941	11	21	1E-05	4E-04
Phenylalanine metabolism	ath00360	15	42	2E-05	5E-04
Proteasome	ath03050	17	58	4E-05	9E-04
Photosynthesis	ath00195	19	77	1E-04	2E-03
RNA polymerase	ath03020	14	45	1E-04	2E-03
Phagosome	ath04145	19	86	3E-04	6E-03
Stilbenoid, diarylheptanoid and gingerol biosynthesis	ath00945	13	46	4E-04	7E-03
Spliceosome	ath03040	32	192	4E-04	8E-03
Biosynthesis of secondary metabolites	ath01110	122	1076	6E-04	0.01
Nicotinate and nicotinamide metabolism	ath00760	7	16	1E-03	0.02
Purine metabolism	ath00230	26	158	2E-03	0.02

Brassinosteroid biosynthesis	ath00905	5	8	2E-03	0.03

Table S7 Annotated DEGs function in photosynthesis pathway

Gene_ID	Gene_name	Product	KEGG K number	Homologous in Arabidopsis
N.20383	atpA.1	ATP synthase CF1 alpha chain	K02111	ArthCp007
N.38609	atpA.2	ATPase subunit alpha	K02111	ArthCp007
N.9727 (CSA029028)	atpA.3	ATPase subunit alpha	K02111	ArthCp007
N.38768	atpF	ATPase subunit b	K02109	ArthCp008
N.39878	atpH	ATPase subunit c	K02110	ArthCp009
N.12825 (CSA020312)	atpI.1	ATPase subunit a	K02108	ArthCp010
N.40468	atpI.2	ATPase subunit a	K02108	ArthCp010
N.34220	petA.1	apocytochrome f precursor	K02634	ArthCp035
N.37107 (CSA013672)	petA.2	apocytochrome f precursor	K02634	ArthCp035
N.48982	petB	cytochrome b6	K02635	ArthCp053
N.1231	petG	cytochrome B6-F complex subunit 5	K02640	ArthCp041
N.1027	psbE	cytochrome b559 alpha subunit	K02707	ArthCp039
N.21333 (CSA015350)	psbC.1	photosystem II CP43 chlorophyll apoprotein	K02705	ArthCp018
N.5683	psbC.2	photosystem II CP43 chlorophyll apoprotein	K02705	ArthCp018
N.5684 (CSA006934)	psbC.3	photosystem II CP43 chlorophyll apoprotein	K02705	ArthCp018
N.21334 (CSA015352)	psbD.1	photosystem II P680 reaction center D2 protein	K02706	ArthCp017
N.5685 (CSA006923)	psbD.2	photosystem II P680 reaction center D2 protein	K02706	ArthCp017
N.33080	psbI	photosystem II protein I	K02710	ArthCp006
N.21083 (CSA031324)	psbK	photosystem II PsbK protein	K02712	ArthCp005

Table S8 Genes encoding transcription factors identified from the in common DEGs

Gene ID	Family	Best hit in A. thaliana	Blast e-value	Description for the best hit

N.26157	C2H2	AT2G28710.1	3.00E-42	C2H2 family protein
N.16335	ERF	AT5G51990.1	7.00E-76	C-repeat-binding factor 4
N.19258	ERF	AT5G47220.1	1.00E-40	ethylene responsive element binding factor 2
N.44348	ERF	AT4G25470.1	4.00E-64	C-repeat/DRE binding factor 2
N.49749	ERF	AT1G68550.1	2.00E-21	ERF family protein
N.33054	HD-ZIP	AT1G69780.1	3.00E-61	HD-ZIP family protein
N.42727 (CSA010605)	NAC	AT5G13180.1	7.00E-26	NAC domain containing protein 83
N.24492 (CSA011152)	NF-YC	AT1G08970.3	5.00E-71	nuclear factor Y, subunit C9
N.2591 (CSA031667)	SBP	AT1G69170.1	6.00E-23	SBP family protein
N.13154	WRKY	AT2G37260.1	2.00E-42	WRKY family protein

Table S9 The differentially expressed genes and their annotation in the spliceosome

Gene_ID	KEGG k number	Gene_name	Enzyme	Homologous in Arabidopsis
N.10935 (CSA011997)	K12836	ATU2AF35 A	U2 snRNP component A	AT1G27650
N.24424 (CSA003395)	K12854	emb1507	U5 small nuclear ribonucleoprotein helicase	AT1G20960
N.17549	K03283	HSC70-1.1	chloroplast heat shock protein 70-1	AT5G02500
N.1471 (CSA016462)	K03283	HSC70-1.2	chloroplast heat shock protein 70-1	AT5G02500
N.46900	K03283	HSC70-1.3	chloroplast heat shock protein 70-1	AT5G02500
N.25443	K03283	HSC70-1.4	chloroplast heat shock protein 70-1	AT5G02500
N.5989 (CSA023075)	K03283	HSC70-1.5	chloroplast heat shock protein 70-1	AT4G24280
N.2454 (CSA021849)	K03283	HSP70.1	heat shock protein 70	AT3G12580
N.44638 (CSA000963)	K03283	HSP70.2	heat shock protein 70	AT3G12580
N.23536	K03283	HSP70.3	heat shock protein 70	AT3G12580
N.47692	K03283	HSP70.4	heat shock protein 70	AT3G12580
N.24172	K03283	HSP70.5	heat shock protein 70	AT3G12580
N.10288	K03283	HSP70.6	heat shock protein 70	AT3G12580
N.37317 (CSA009227)	K03283	HSP70.7	heat shock protein 70	AT3G12580
N.47270	K03283	HSP70.8	heat shock protein 70	AT3G12580
N.1400 (CSA027456)	K04043	MTHSC70-2	mitochondrial HSO70 2	AT5G09590

N.9047 (CSA035886)	K12891	SC35	splicing factor, arginine/serine-rich 2	AT5G64200
N.53682	K12891	SC35	splicing factor, arginine/serine-rich 2	AT5G64200
N.49039 (CSA013761)	K12741	UBA2A	Common spliceosomal components	AT3G56860
